# One-pot synthesis of dicyclopenta-fused peropyrene via a fourfold alkyne annulation

**DOI:** 10.3762/bjoc.16.72

**Published:** 2020-04-20

**Authors:** Ji Ma, Yubin Fu, Junzhi Liu, Xinliang Feng

**Affiliations:** 1 Center for Advancing Electronics Dresden (cfaed) & Faculty of Chemistry and Food Chemistry, Technische Universität Dresden, 01062 Dresden, Germany; 2 Department of Chemistry and State Key Laboratory of Synthetic Chemistry, The University of Hong Kong, Pokfulam Road, Hong Kong, China

**Keywords:** alkyne annulation, cyclopenta-fused polycyclic aromatic hydrocarbons, nonplanarity, peropyrene, regioselectivity

## Abstract

A novel dicyclopenta-fused peropyrene derivative **1** was synthesized via a palladium-catalyzed four-fold alkyne annulation of 1,3,6,8-tetrabromo-2,7-diphenylpyrene (**5**) with diphenylacetylene. The annulative π-extension reaction toward **1** involved a twofold [3 + 2] cyclopentannulation and subsequent twofold [4 + 2] benzannulation. The structure of **1** is unambiguously confirmed by X-ray crystallography; **1** adopted a twisted geometry due to the steric hindrance of the phenyl rings and the hydrogen substituents at the bay regions. Notably, compound **1** exhibits a narrow energy gap (1.78 eV) and a lower LUMO energy level than the parent peropyrene without the fusion of the five-membered rings. In addition, the effects of the *peri*-fused pentagons on the aromaticity and molecular orbitals of **1** were evaluated by theoretical calculations. This work presents an efficient method to develop π-extended aromatic hydrocarbons with cyclopenta moieties.

## Introduction

**Scheme 1:**
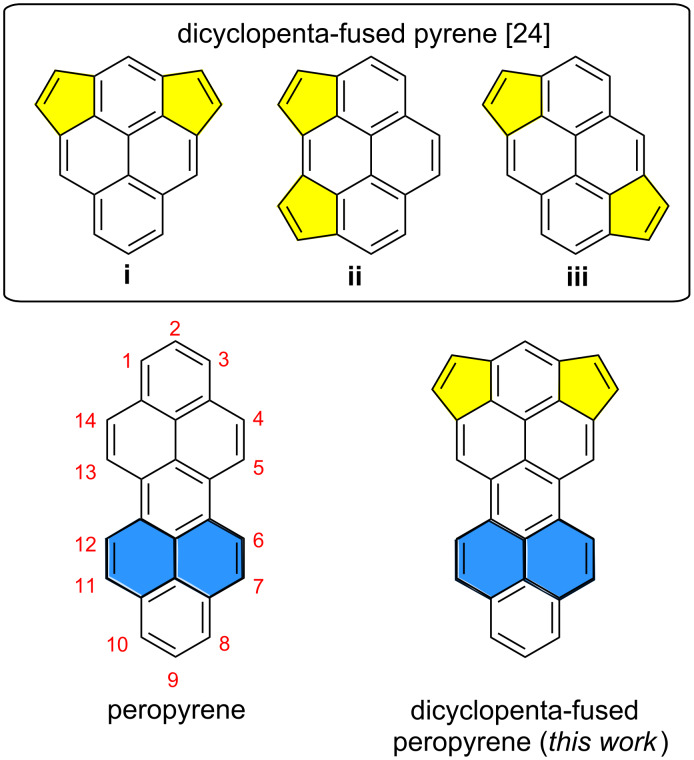
Chemical structures of dicyclopenta-fused pyrene derivatives **i**–**iii**, peropyrene and the dicyclopenta-fused peropyrene reported in this work.

Significant efforts have been recently devoted to the synthesis of nonalternant cyclopenta-fused polycyclic aromatic hydrocarbons (CP-PAHs), which represent the topological subunits of fullerenes and exhibit high chemical, physical and biological activities [1–10]. Thanks to development in organic synthetic methodology, CP-PAHs with peripheral pentagons could be realized [11–17]. Among them, the cyclopenta-fused pyrenes are an important class of CP-PAHs owing to their unique physical and photophysical properties, such as high electron affinities and anomalous fluorescence [17–20]. However, the reported synthetic methods towards the (di-)cyclopenta-fused pyrene congeners (**i**–**iii**, Scheme 1) have mainly been reliant on the flash vacuum pyrolysis of suitable precursors under harsh conditions (*T* ≥ 900 °C), which resulted in relatively low yields [21–24]. Palladium-catalyzed annulation has been recently proven as an efficient route to get access to aromatic hydrocarbons with *peri*-fused five-membered rings [25–27]. For instance, the dicyclopenta-fused pyrene derivatives **ii** and **iii** (Scheme 1) were successfully synthesized through palladium-catalyzed carbannulation of brominated pyrene with arylacetylenes in good yield [28–29]. However, the larger CP-PAHs beyond the pyrene core, or its extended analogs [30] remain elusive. Peropyrene (Scheme 1), as the higher homolog of pyrene, has recently attracted attention because of its promising applications in optoelectronics, e.g., for singlet fission materials [31–33]. However, the synthesis of cyclopenta-fused aromatics based on peropyrene has never been achieved due to the lack of suitable synthetic protocols.

**Scheme 2:**
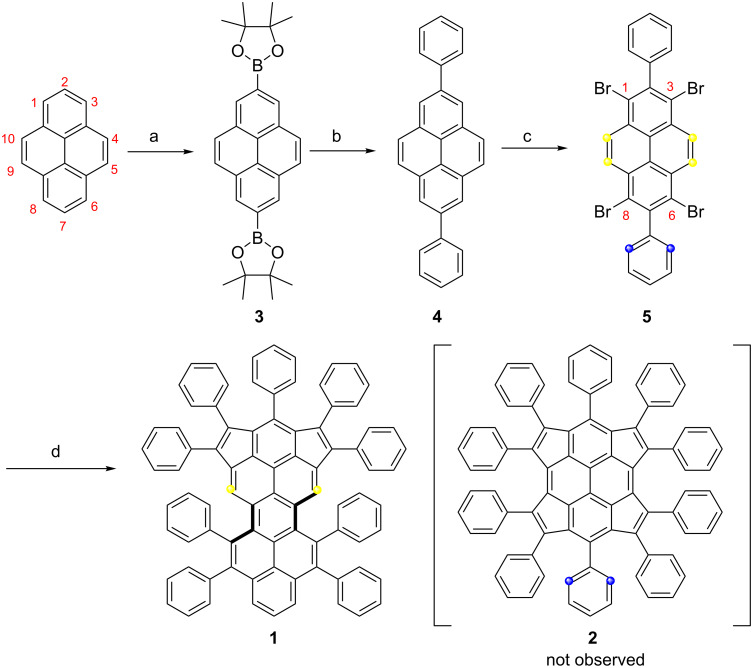
Synthetic route towards compound **1**. a) B_2_pin_2_, dtbpy, [Ir(OMe)cod]_2_, cyclohexane, 70 °C, 20 h, 67%; b) Pd(PPh_3_)_4_, bromobenzene, Na_2_CO_3_, toluene/EtOH/H_2_O, Aliquit 336, 90 °C, 48 h, 77%; c) Br_2_, nitrobenzene, 120 °C, 5 h, 86%; d) 1,2-diphenylethyne, Pd_2_(dba)_3_, P(*o*-tol)_3_, KOAc, LiCl, DMF, 130 °C, microwave, 6 h, 5%.

In this work, while aiming at the synthesis of the novel tetracyclopenta-fused pyrene derivative **2** through the quadruple annulation of 1,3,6,8-tetrabromo-2,7-diphenylpyrene (**5**) with 1,2-diphenylethyne, an unprecedent dicyclopenta-fused peropyrene congener **1** was obtained (Scheme 2). Interestingly, from the single-crystal analysis, compound **1** shows slight twisting of the peropyrene core with an overall end-to-end twist angle of 21.4° as a result of the steric repulsion at the bay positions. Compared to the parent peropyrene, the pentagon-annulated derivative **1** possesses a narrow optical energy gap (1.78 eV) and displays an efficient highest occupied molecular orbital (HOMO)–lowest unoccupied molecular orbital (LUMO) separation.

## Results and Discussion

**Figure 1:**
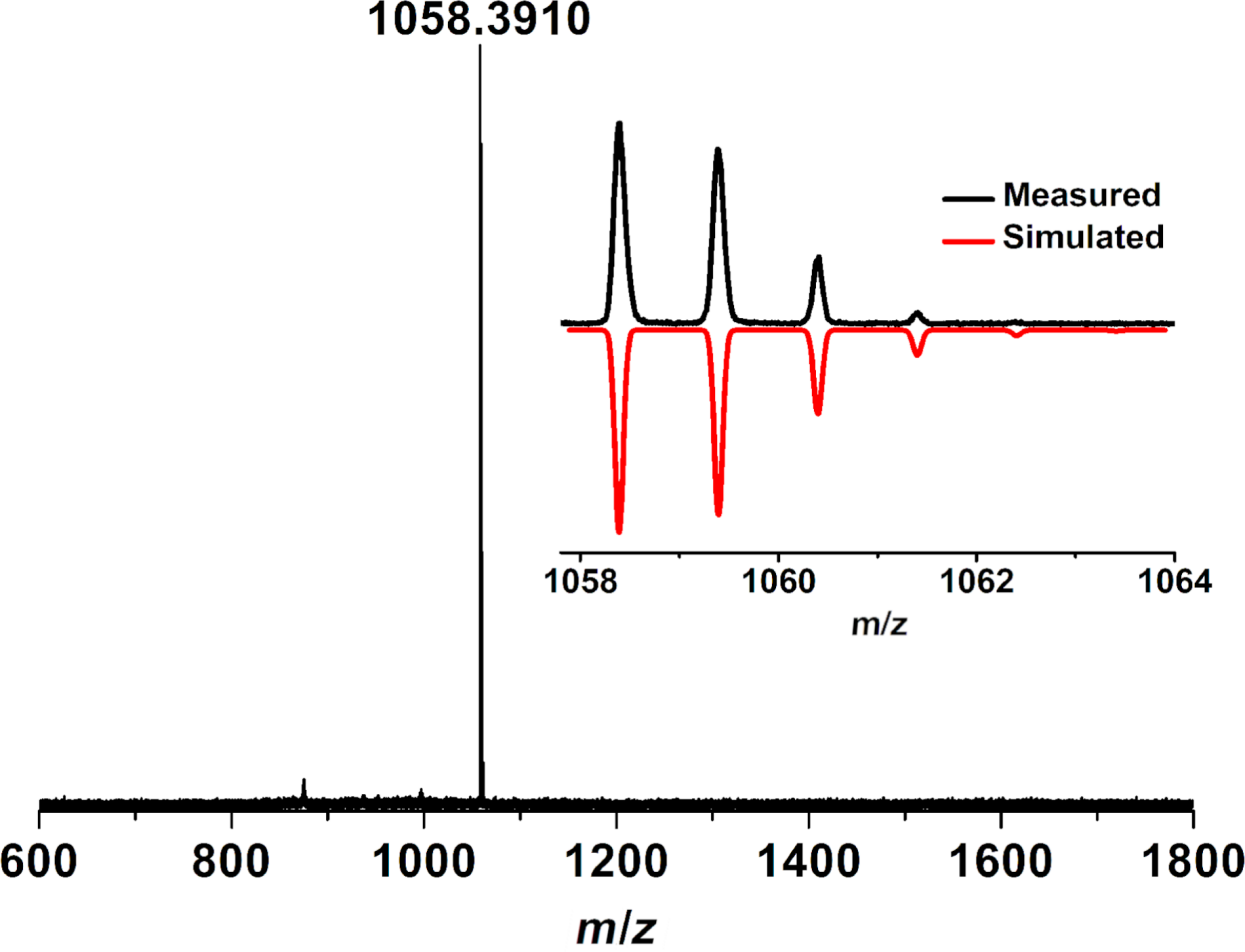
High-resolution MALDI-TOF mass spectrum of **1**. Inset: isotopic distribution compared to mass spectrum simulated for C_84_H_50_.

The synthesis of compound **1** is depicted in Scheme 2. Firstly, 2,7-bis(Bpin)pyrene (**3**) was prepared using an iridium-catalyzed borylation of pyrene (67% yield). Then, 2,7-diphenylpyrene (**4**) was obtained by Suzuki cross-coupling of **3** and bromobenzene in 77% yield. After that, the selective bromination of **4** with 4.4 equiv of bromine in nitrobenzene solution at 120 °C afforded 1,3,6,8-tetrabromo-2,7-diphenylpyrene (**5**) in excellent yield (86%). Compared to insoluble 1,3,6,8-tetrabromopyrene [34], the diphenyl-substituted compound **5** exhibited excellent solubility in common organic solvents, such as dichloromethane, chloroform, tetrahydrofuran and toluene, allowing a full characterization by NMR analyses. Finally, the palladium-catalyzed cyclopentannulation of compound **5** with 1,2-diphenylethyne under microwave conditions using the catalyst system of [Pd_2_(dba)_3_] and P(*o*-tol)_3_ afforded a dark red solid in 5% yield after purification. The obtained product showed an intense peak at 1058.3910 during MALDI–TOF mass analysis (positive mode, dithranol as the matrix) that matched well with the expected molecular mass of *m*/*z* 1058.3913 (calcd for C_84_H_50_: [M]^+^) for dicyclopenta-fused peropyrene **1**. Furthermore, the observed isotopic distribution was fully consistent with its simulated spectrum (Figure 1). Characterization of the resultant product by single crystal X-ray analysis unambiguously revealed the selective formation of **1** through twofold [3 + 2] pentannulation and sequent twofold [4 + 2] benzannulation, instead of the desired tetracyclopenta[*cd*,*fg*,*jk*,*mn*]pyrene (**2**). The selective formation of **1** could be rationalized by the steric hindrance of the phenyl rings after the twofold [3 + 2] alkyne pentannulated intermediate (Scheme S2, Supporting Information File 1), and the sequent annulation was favorable for the formation of six-membered rings. Nevertheless, the existence of several rotamers of **1** derived from the restricted rotation of the peripheral phenyl ring substituents and its nonplanar geometry prevented the structure elucidation by proton NMR analysis [35].

**Figure 2:**
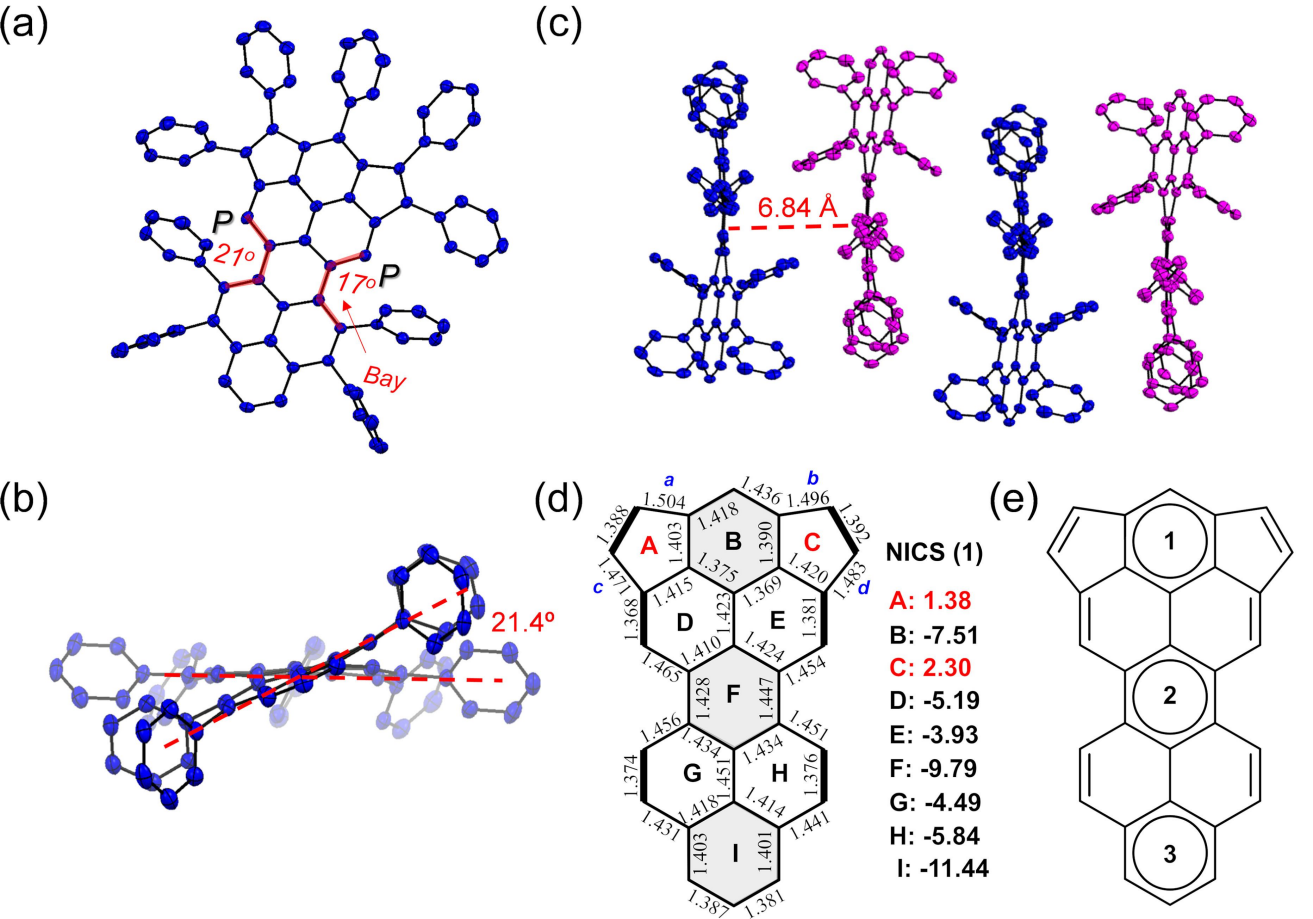
Single-crystal X-ray structure of **1**. (a) Top view and (b) side view of the (*P*,*P*) isomer. c) Crystal packing of the enantiomer pairs (*P*,*P* and *M*,*M*) of **1**. Hydrogen atoms and solvent molecules are omitted for clarity. (d) Selected bond lengths (from the crystal structure) and calculated NICS(1) values of rings A–I in **1**. (e) Clar valence structure representation of **1** with three benzeneoid rings.

Single crystals of **1** were obtained by slow evaporation from a carbon disulfide solution, allowing us to disclose the molecular structure by X-ray crystallography (Figure 2a). As shown in Figure 2a, the crystal structure of **1** clearly displayed a nonplanar conformation, resulting from steric repulsion between the phenyl groups and the hydrogen atoms at the bay positions. Interestingly, the splay angle of each bay position showed slight differences with a value of 17° and 21°, respectively. This contortion resulted in a molecular backbone with an overall end-to-end twist angle of 21.4° (Figure 2b), which is slightly larger than that of the reported 5,13-diphenylperopyrene derivative (18°) [32]. The twisted carbon skeleton of **1** makes it a chiral molecule with enantiomers (*P*,*P*) and (*M*,*M*) configuration in the packing mode through a face-to-face slip-stacking arrangement, with a minimum interplanar spacing of 6.84 Å (Figure 2c). In addition, the C–C bond lengths in **1** are shown in Figure 2d. The short lengths of the black bold bonds (1.368–1.392 Å) in **1** suggested their double bond character (C=C is typically 1.337 Å). These results are in good agreement with the resonance structure of **1** that is assigned by Clar’s aromatic sextet theory (Figure 2e). Interestingly, the long bond length of *a*, *b*, *c*, and *d* (1.471–1.504 Å) indicated that the double bonds on the five-membered rings have a small contribution to the overall aromatic delocalization of the carbon framework [26]. In order to evaluate the aromaticity of **1**, a nucleus-independent chemical shift (NICS) calculation was conducted. As shown in Figure 2d, the positive NICS(1) values of the five-membered rings A and C reveal the slightly anti-aromatic feature. The rings B, F and I appear to have more aromatic character, while the rings D, E, G and H become less aromatic, which is in accordance with the resonance structure of **1** as shown in Figure 2e.

**Figure 3:**
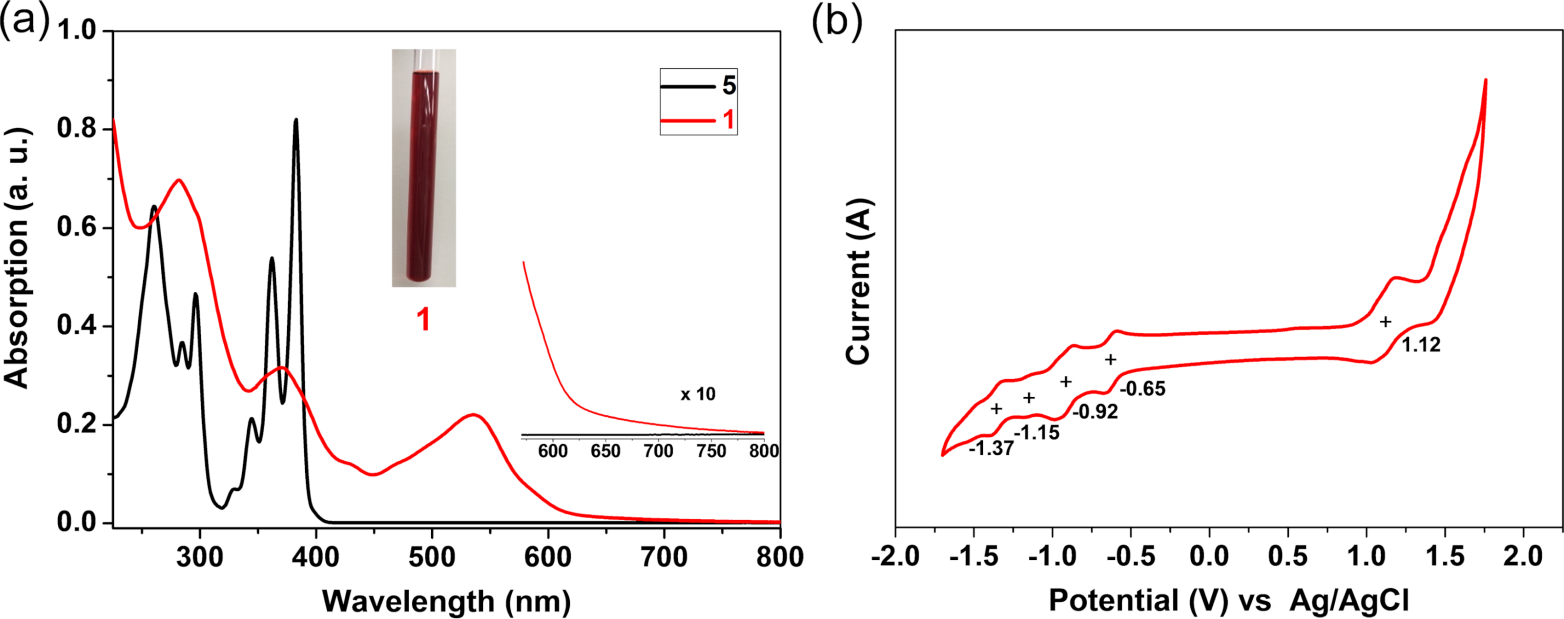
(a) UV–vis absorption spectra of precursor **5** and **1** in CH_2_Cl_2_ solution (10^−5^ M). Inset: photograph of a CH_2_Cl_2_ solution of **1**. (b) Cyclic voltammogram of **1** (0.1 M *n*-Bu_4_NPF_6_ in DCM) at a scan rate of 50 mV s^−1^.

The UV–vis absorption spectra of compounds **5** and **1** in DCM are compared in Figure 3a. The maximum absorption peak of **1** is significantly red-shifted compared to that of precursor **5**, which can be attributed to the extended conjugation of **1** after the annulation. Compound **1** shows a broad absorption band in the range of 449–690 nm with the absorption maximum at 537 nm, which also displays a large red-shift (70 nm) compared with the reported peropyrene derivative [32]. The optical energy gap of **1** is determined to be 1.78 eV from the onset of its UV–vis absorption spectrum. Similar to the cyclopenta-fused pyrene derivatives [28–29], compound **1** does not show detectable fluorescence emission. Furthermore, the electrochemical properties of **1** was probed by cyclic voltammetry (CV) in DCM (Figure 3b). According to the CV analysis, compound **1** exhibits one reversible oxidation wave with half-wave potentials (*E*_1/2_^ox^) at 1.12 V and four reduction waves with half-wave potentials (*E*_1/2_^red^) at −0.65, −0.92, −1.15, and −1.37 V (vs Ag/AgCl). The HOMO/LUMO energy levels are estimated to be −5.37 /−3.80 eV, respectively, based on the onset potentials of the first oxidation/reduction waves. Accordingly, the corresponding electrochemical energy gap (*E*_g_^EC^) of **1** is derived to be 1.57 eV, which is slightly smaller than the optical energy gap (1.78 eV).

**Figure 4:**
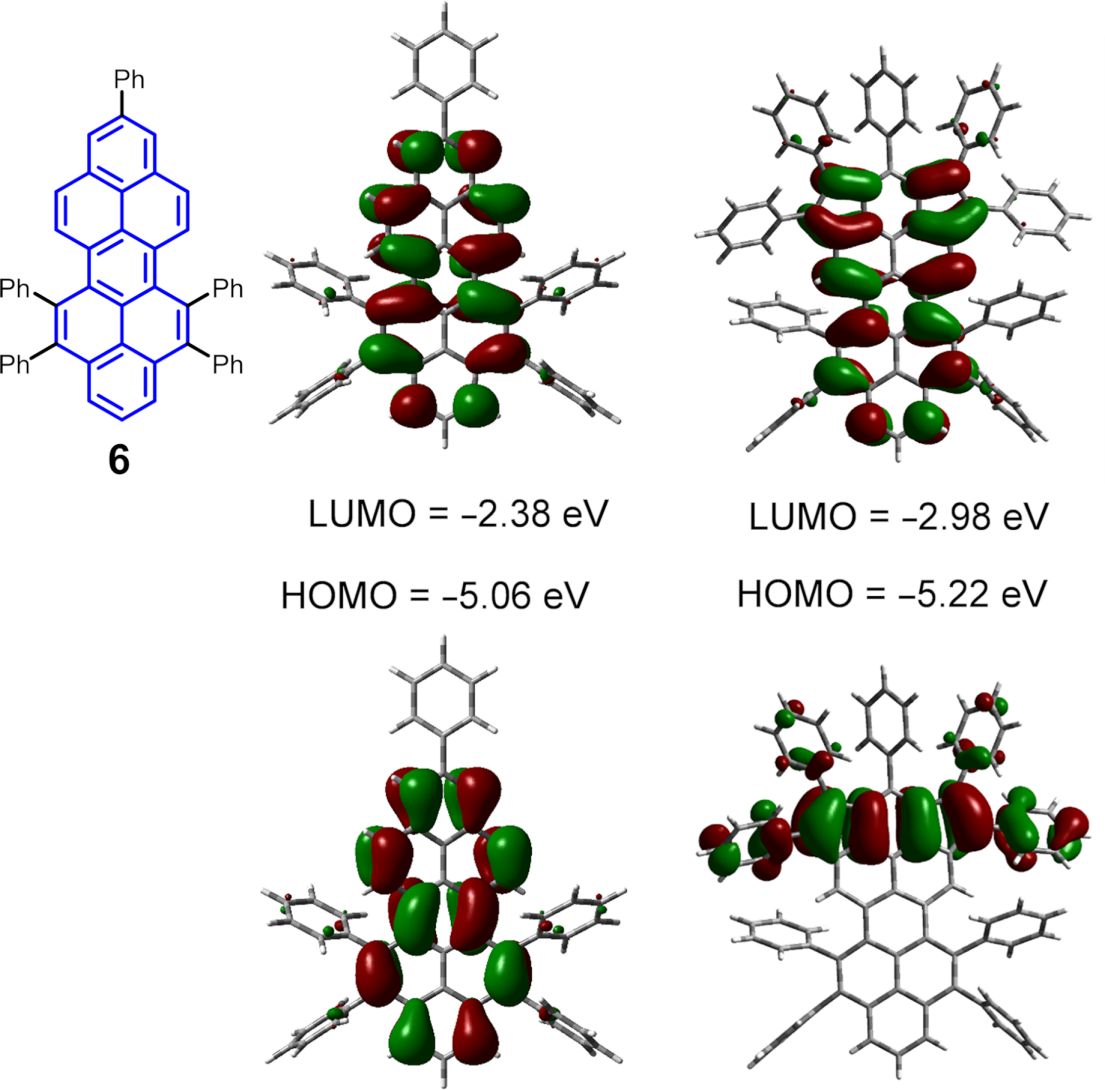
Molecular orbitals of peropyrene derivative **6** and the dicyclopenta-fused peropyrene **1**.

In order to gain a deeper insight into the effects of the fused 5-membered rings on the peropyrene core, the electronic structures and the frontier orbitals of the peropyrene derivative **6** without pentagons and of compound **1** are compared by DFT calculations at the B3LYP/6-311++G(d,p) level. As shown in Figure 4, the LUMO and HOMO of **6** are both delocalized over the aromatic core. In contrast to **6**, compound **1** presents a significant difference in the shape of its molecular orbitals. The LUMO of **1** is mainly localized on the core, whereas the HOMO keeps a line of high electron density along the fused five-membered rings (rings A and C in Figure 2d) and the central six-membered ring B. The large difference between the LUMO and HOMO leads to an intramolecular charge transfer, resulting in broad absorption bands in the UV–vis spectrum (Figure 3a) [36]. In addition, the LUMO energy of **1** (−2.98 eV) is significantly lower than that of **6** (−2.38 eV), which is responsible for the narrower energy gap of **1** (2.24 eV).

## Conclusion

In summary, we demonstrated the first synthesis and characterization of a dicyclopenta-fused peropyrene **1** starting from pyrene in four steps in which the twofold pentannulation and subsequent twofold benzannulation based on 1,3,6,8-tetrabromo-2,7-diphenylpyrene is the key step. The single crystal X-ray diffraction analysis revealed a twisted structure of **1** due to the steric hindrance at the bay positions. From the bond length analysis and DFT calculations, CP-PAH **1** consists of the aromatic peropyrene core with two slightly antiaromatic *peri*-fused five-membered rings. In addition, dicyclopenta-fused peropyrene **1** possesses a decreased LUMO energy level compared to the parent peropyrene without five-membered rings, which is responsible for the resultant low energy gap (1.78 eV). This work report herein paves the way toward the synthesis of novel cyclopenta-fused PAHs in large π-systems.

## Supporting Information

File 1 Experimental details, synthetic procedures, single crystal X-ray data for **1**, detailed theoretical calculations, and analytical data for the compounds.
