# Supplementary material for: One-pot synthesis of dicyclopenta-fused peropyrene via a fourfold alkyne annulation
Source: Beilstein J Org Chem. 2020 Apr 20;16:791–7. doi: 10.3762/bjoc.16.72 (PMC7188986; doi:10.3762/bjoc.16.72)
Supplement: File 1 — Experimental details, synthetic procedures, single crystal X-ray data for 1, detailed theoretical calculations, and analytical data for the compounds. [file Beilstein_J_Org_Chem-16-791-s001.pdf]

BEILSTEIN JOURNAL OF ORGANIC CHEMISTRY

Supporting Information

for

One-pot synthesis of dicyclopenta-fused peropyrene via a fourfold alkyne annulation

Ji Ma, Yubin Fu, Junzhi Liu and Xinliang Feng

Beilstein J. Org. Chem. **2020,** *16,* 791–797. doi:10.3762/bjoc.16.72

Experimental details, synthetic procedures, single crystal X-ray data for 1, detailed theoretical calculations, and analytical data for the compounds

License and Terms: This is a supporting information file under the terms of the Creative Commons Attribution License (http://creativecommons.org/licenses/by/4.0). Please note that the reuse, redistribution and reproduction in particular requires that the authors and source are credited.
The license is subject to the *Beilstein Journal of Organic Chemistry* terms and conditions: (https://www.beilstein-journals.org/bjoc)

Table of Content

1. Experimental section

   1.1 General methods and materials

   1.2 Detailed synthetic procedure and characterization data

   1.3 Proposed pathway for the annulative π-extension

2. X-ray crystallographic analysis of **1**

3. Details of the theoretical calculations

4. NMR spectra

5. MALDI-TOF mass spectra

6. References

1. Experimental section

1.1 General methods and materials

All reagents were purchased from commercial suppliers and used as received without further purification. The ^{1}H NMR and ^{13}C NMR spectra were recorded in deuterated solvents on a Bruker DPX 300 or an AV-III 600 NMR spectrometer. Chemical shifts are given in ppm relative to tetramethylsilane (TMS), coupling constants *J* are given in Hertz, the solvent signals were used as reference. Coupling constants were determined assuming first-order spin-spin coupling. The following abbreviations were used to explain the multiplicities: s = singlet, d = doublet, t = triplet, m = multiplet. Matrix-assisted laser desorption/ionisation time-of-flight (MALDI-TOF) mass spectra were recorded on a Bruker Autoflex Speed instrument (Bruker Daltonics, Bremen, Germany) using 1.8-dihydroxyanthrone (dithranol) or *trans*-2-[3-(4-*tert*-Butylphenyl)-2-methyl-2-propenylidene]malononitrile (DCTB) as matrix substrate. Preparative recycling gel permeation chromatography (r-GPC) was performed with a JAI LC-91XX II NEXT instrument equipped with JAIGEL-1H/JAIGEL-2H columns using chloroform as an eluent. High-performance liquid chromatography (HPLC) was performed with a Shimadzu HPLC instrument equipped with a silica column using toluene/hexane (1:3) as an eluent. UV–visible (UV) spectra were measured on an Agilent Cary 5000 UV-VIS-NIR spectrophotometer by using a 10 mm optical path quartz cell at room temperature. Cyclic voltammetry (CV) measurements were carried out on a CHI 760 E potentiostat (CH Instruments, USA) in a three-electrode cell in a dichloromethane solution of Bu_4NPF_6 (0.1 M) with a scan rate of 50 mV/s at room temperature. A Pt wire, Ag/AgCl (3 M KCl solution), and a glassy carbon electrode were used as the counter electrode, the reference electrode, and the working electrode, respectively.

1.2 Detailed synthetic procedure and characterization data

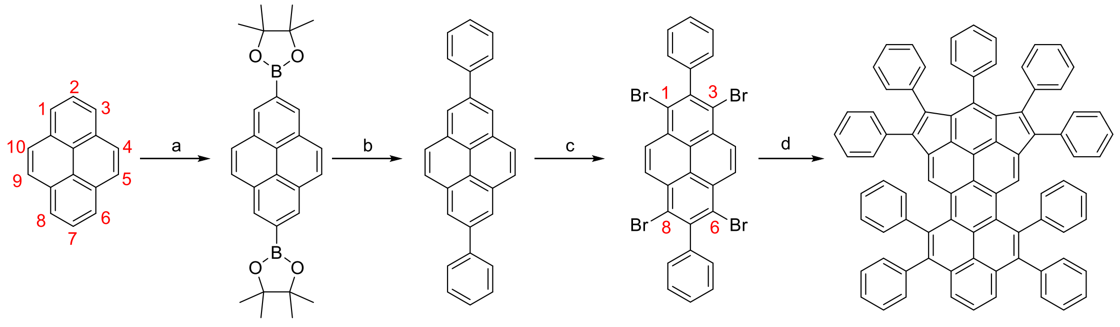

Scheme S1. Synthetic route to **1**. (a) B_2pin_2, dtbpy, $[Ir(OMe)Cod]_2$, cyclohexane, 70 °C, 20 h,

67%; (b) $Pd(PPh_3)_4$, bromobenzene, Na_2CO_3, toluene/EtOH/H_2O, Aliquit 336, 90 °C, 48 h, 77%; (c) Br_2, nitrobenzene, 120 °C, 5 h, 86%; (d) 1,2-diphenylethyne, $[Pd_2(dba)_3]$, $P(o\text{-}Tol)_3$, KOAc, LiCl, DMF, 130 °C, microwave, 6 h, 5%.

2,7-Bis(4,4,5,5-tetramethyl-1,3,2-dioxaborolan-2-yl)pyrene (3)

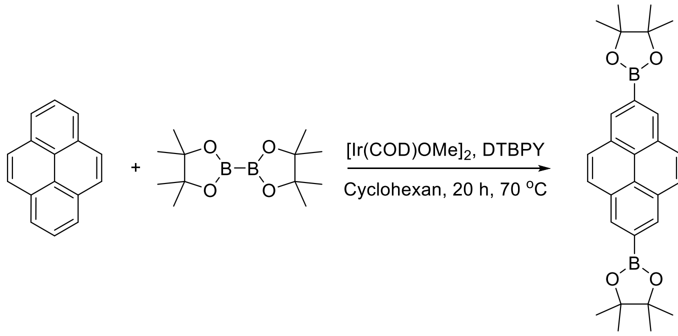

Compound **3** was synthesized according to the reported method [1]. Pyrene (4 g, 19.78 mmol), bispinacolatodiborane (10.55 g, 41.54 mmol), $[Ir(OMe)COD]_2$ (500 mg, 0.75 mmol) and 4,4'-di-*tert*-butyl-2,2'-bipyridine (550 mg, 2.05 mmol) were placed in a 250 mL flame dried Schlenk flask. Degassed dry cyclohexane (90 ml) was added. The solution was stirred at 70 °C for 20 h under an argon atmosphere. After cooling to room temperature the mixture was filtered through a silica plug (the plug was flushed with Dichloromethane). The solvent was evaporated and the residue was purified by column-chromatography (SiO_2; gradient DCM/Hex 1:2 to DCM) to afford compound **3** as a white solid (6 g, 67%). ^{1}H NMR (300 MHz, $CDCl_3$) δ: 8.59 (s, 2H), 8.05 (s, 2H), 1.42 (s, 12H). ^{13}C NMR (75 MHz, $CDCl_3$) δ: = 131.18, 130.87, 127.62, 126.29, 84.15, 24.98. HR-MALDI-TOF: found for $[M]^+$: 454.2487, calcd. for $[M]^+$: 454.2487 (deviation: 0 ppm).

2,7-Diphenylpyrene (4)

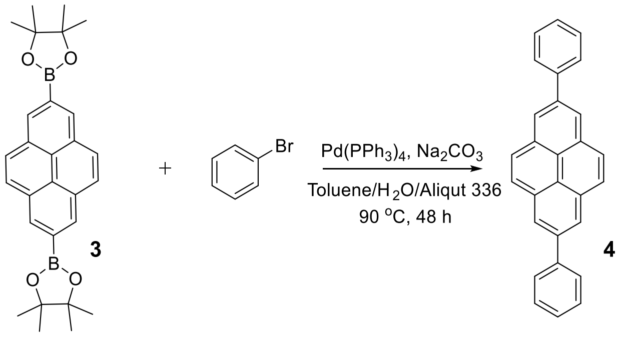

The synthetic procedure for **4** is in a similar manner as described in ref. [2]. To a solution of compound **3** (2.0 g, 4.40 mmol) and the corresponding bromobenzene (4.6 mL, 44.0 mmol)

dissolved in toluene (60 mL) was added a 2 M solution of sodium carbonate (2.3 g, 22.0 mmol) dissolved in water (10 mL). The mixture was bubbled with Argon for 30 min. Then, tetrakis(triphenylphosphine)palladium(0) (508 mg, 0.44 mmol) and phase-transfer agent Aliquat 336 (5 drops) were added. The mixture was heated to 90 °C for 48 h under argon atmosphere. The resulting mixture was cooled to room temperature, most of the solution was removed by rotavapor and then poured into methanol (300 mL). The resulting precipitate was filtered off, washed with water, dilute acid (5% HCl), water, methanol, then with acetone to remove the starting material as well as the mono-substituted by-product. After drying in vacuum, compound **4** is obtained as a white solid (1.2 g, 77%). ^{1}H NMR (300 MHz, $CDCl_3$) δ: 8.34 (s, 4H), 8.12 (s, 4H), 7.88 (d, *J*=9 Hz, 4H), 7.53 (t, *J*=6 Hz, 4H), 7.41 ppm (t, *J*=6 Hz, 2H); ^{13}C NMR (75 MHz, $CDCl_3$) δ: 141.51, 138.97, 131.53, 128.99, 128.06, 127.94, 127.44, 123.90, 123.78. HR-MALDI-TOF: found for $[M]^+$: 354.1411, calcd. for $[M]^+$: 354.1409 (deviation: 0.5 ppm).

1,3,6,8-Tetrabromo-2,7-diphenylpyrene (5)

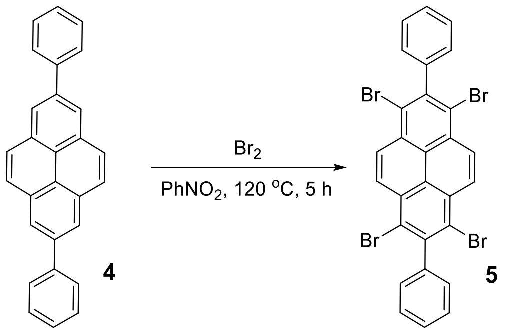

The synthetic procedure for **5** is in a similar manner as described in ref. [3]. A mixture of bromine (0.13 mL, 2.46 mmol) with nitrobenzene (3 mL) was added dropwise, with vigorous stirring, to a solution of compound **4** (200 mg, 0.56 mmoL) in nitrobenzene (8 mL) at 120 °C. The mixture was kept at 120 °C for 5 h and then cooled to room temperature. The cooled reaction solution was poured into methanol (150 mL) and the precipitate filtered off. Further drying of the precipitate in high vacuum gave the crude product. Then it was purified by recrystallization from chloroform to obtain the title compound in a yield of 86% (320 mg). ^{1}H NMR (600 MHz, $CDCl_3$) δ: 8.69 (s, 4H), 7.58 (t, *J*=6 Hz, 4H) , 7.53 (t, *J*=12 Hz, 2H), 7.37 (d, *J*=12 Hz, 4H); ^{13}C NMR (151 MHz, $CDCl_3$) δ: 143.41, 143.13, 129.47, 129.32, 129.07, 128.40, 128.21, 124.61, 123.24. HR-MALDI-TOF: found for $[M]^+$: 665.7798, calcd. for $[M]^+$: 665.7829 (deviation: -4.6 ppm).

1,2,3,4,5,7,8,12,13-Nonaphenylbenzo[*lm*]-s-indaceno[1,8,7-*bcde*]perylene (1)

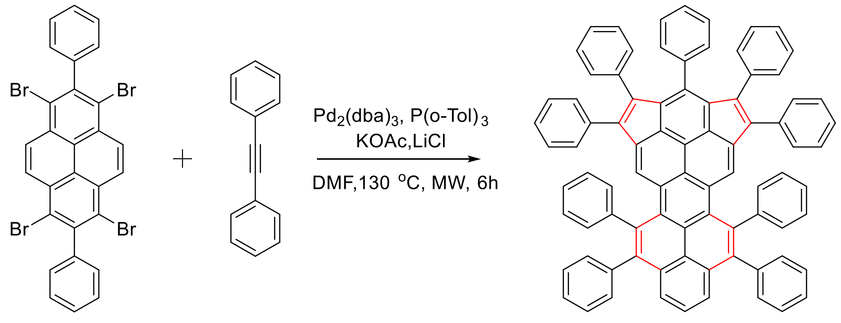

5 **1**

In a glove box, the starting material **5** (50 mg, 0.075 mmol), diphenyl acetylene (0.375 mmol, 66.8 mg), $Pd_2(dba)_3$ (13 mg, 20 mmol %), P(*o*-Tol)$_3$ (9 mg, 30 mmol %), KOAc (110 mg, 1.125 mmol), LiCl (19 mg, 0.45 mmol) and dry DMF (4 mL) were combined into an oven-dried 10 mL glass reaction vail. The vial was sealed, evacuated, purged with nitrogen and heated in a CEM Discovery microwave reactor at 130 °C for 6 h. The reaction mixture was cooled to room temperature and poured into 50 mL methanol and filtered. The residue was washed with methanol to obtain a red/brown solid which was then pre-purified by r-GPC (chloroform as eluent). Subsequently, the fraction containing the target molecular weight from r-GPC (checked by MALDI-TOF MS) was then subjected to reverse phase HPLC with silica column (20 × 250 mm) using toluene/hexane (1:3) as the mobile phase, which gave the unexpected compound **1** (3.9 mg, 5%). HR-MALDI-TOF: found for $[M]^+$: 1058.3910, calcd. for $[M]^+$: 1058.3913 (deviation: - 0.2 ppm).

1.3 Proposed pathway of the annulation reaction towards 1

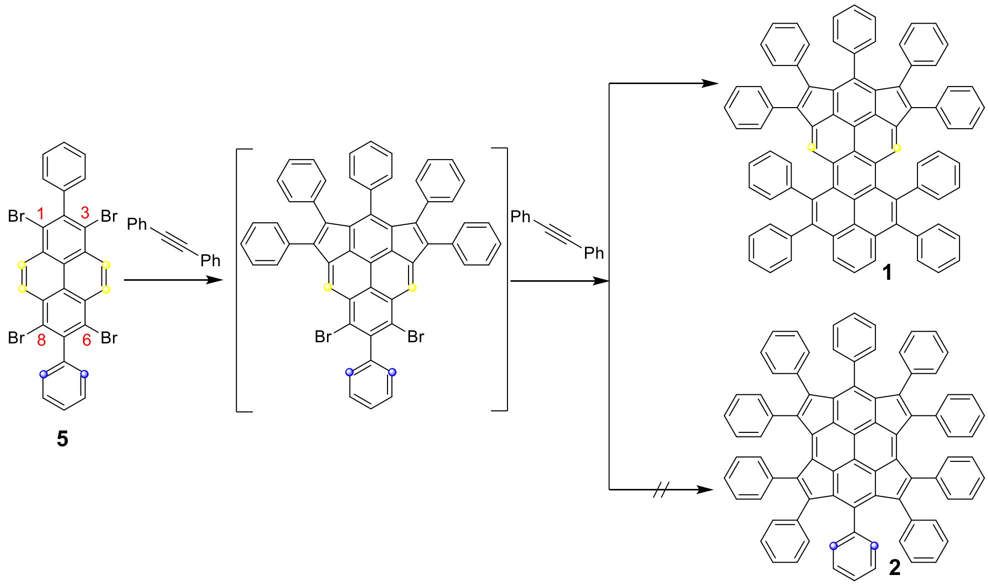

Scheme S2. Possible pathway to the formation of **1** from the precursor **5**.

2. X-ray crystallographic analysis of 1

Single crystals of **1** were obtained by slowly evaporation from a carbon disulfide solution. The X-ray crystallographic coordinates for structures reported in this article have been deposited at the Cambridge Crystallographic Data Centre (CCDC), under deposition number CCDC 1984017 (**1**). These data can be obtained free of charge from CCDC via http://www.ccdc.cam.ac.uk/data_request/cif

Table **S1**. Summary of crystal data and reflection collection parameters for compound **1**

	**1**
Moiety formula	$C_{84}H_{50}$, $2(CS_2)$
Formula weight	1211.50
space group	*P*-1
a, Å	12.5707(6)
b, Å	14.6611(8)
c, Å	17.6163(12)
α, deg	67.575(6)
β, deg	85.656(5)
γ, deg	86.344(4)
V, Å^3	2990.4 (3)
Z	2
$D_{calcd.}$, g cm^{-3}	1.345
F_{000}	1260.0
Temperature, K	100 (10)
Radiation	CuKα (λ = 1.54184)
μ, mm^{-1}	1.849
2Θ range for data collection/°	125.448
h, k, l max	14, 16, 20
no. of reflections	9318
Tmin, Tmax	0.646, 1.000
R_1, wR_2 (reflections)	0.0836 (6314), 0.2654 (9318)
Goodness-of-fit on F^2	1.028

3. Details of the theoretical calculations

All density functional theory (DFT) calculations were performed using the Gaussian 09 program [4]. The B3LYP functional was used for geometry optimization in the ground state. The 6-311+G(2d, p) basis set was used for the C and H atoms. All the geometry optimization was done in the gas phase and based on the single crystal structure. Nucleus independent chemical shifts (NICS) values were calculated using the standard gauge invariant atomic orbital (GIAO) [5] method at B3LYP functional.

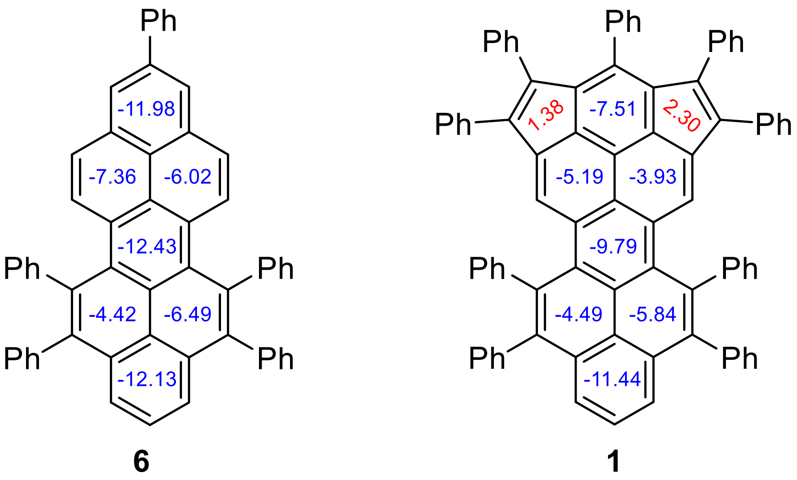

Figure S1. The NICS(1) values of the individual rings in **6** and **1**, calculated at the GIAO-B3LYP/6-311+G(2d,p) level of theory.

4. NMR characterization

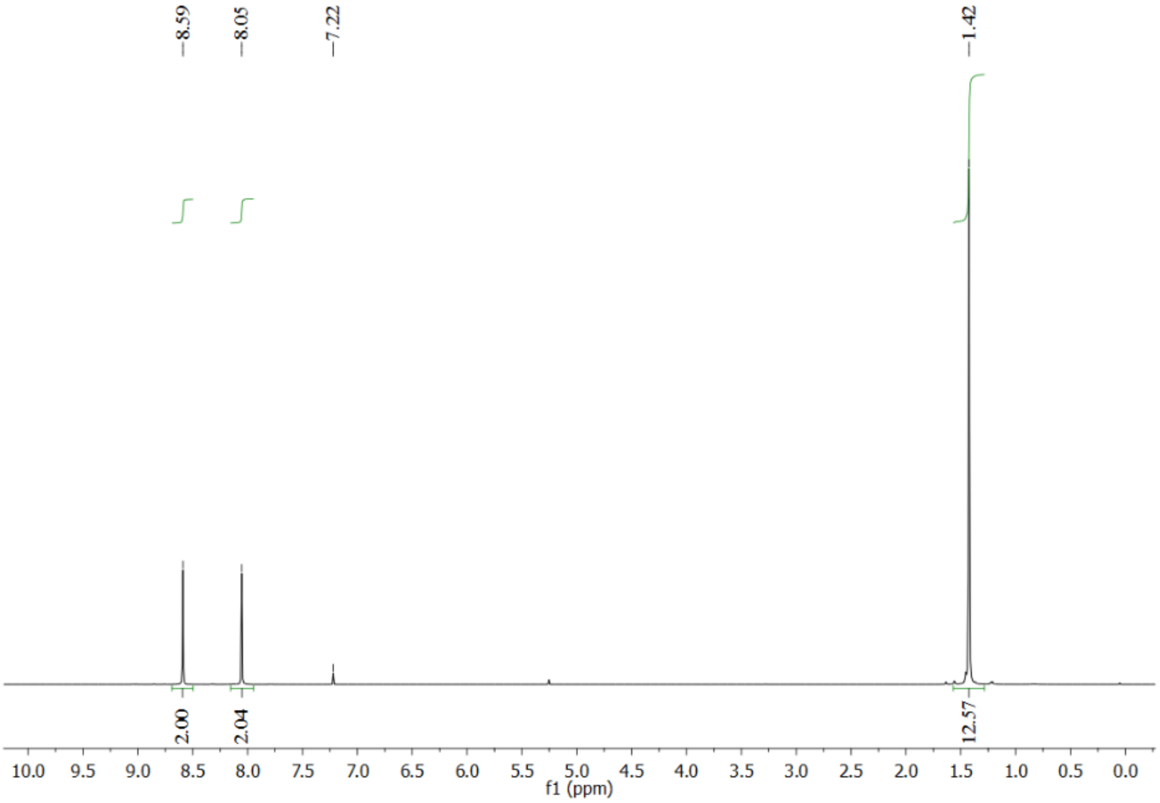

Figure S2. ^{1}H NMR spectrum of compound **3** (300 MHz, $CDCl_3$, rt).

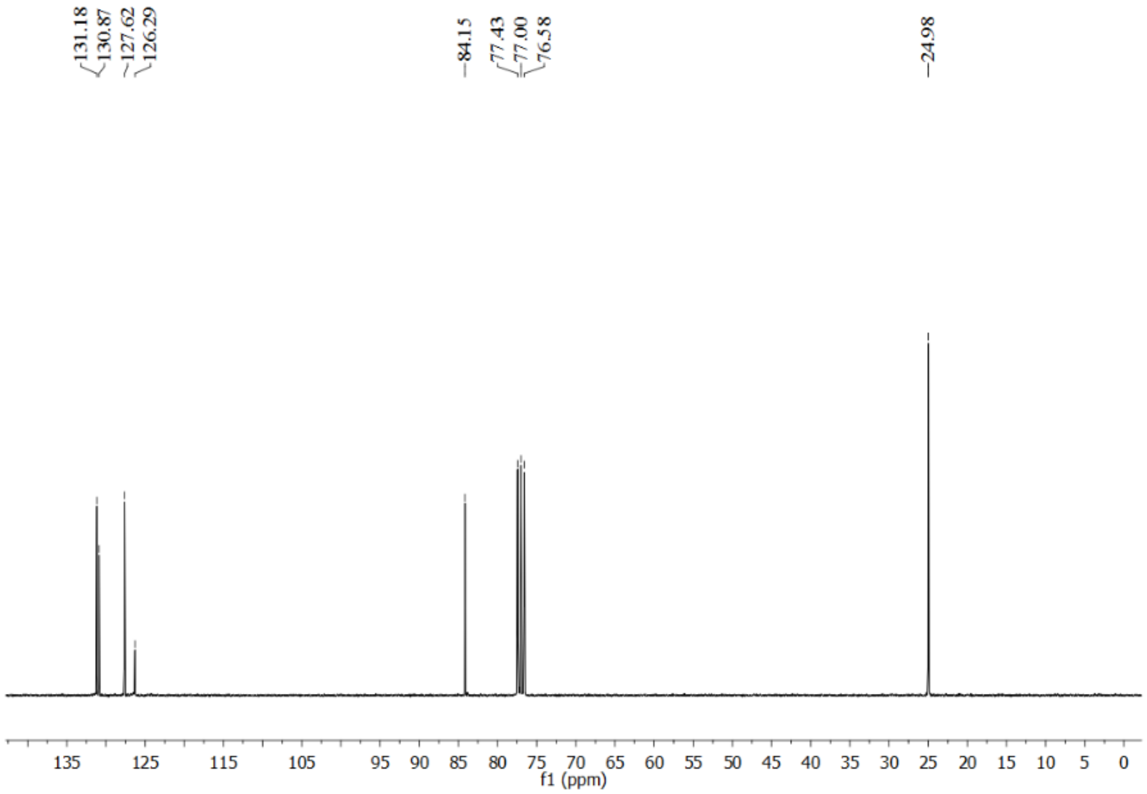

Figure S3. ^{13}C NMR spectrum of compound **3** (75 MHz, $CDCl_3$, rt).

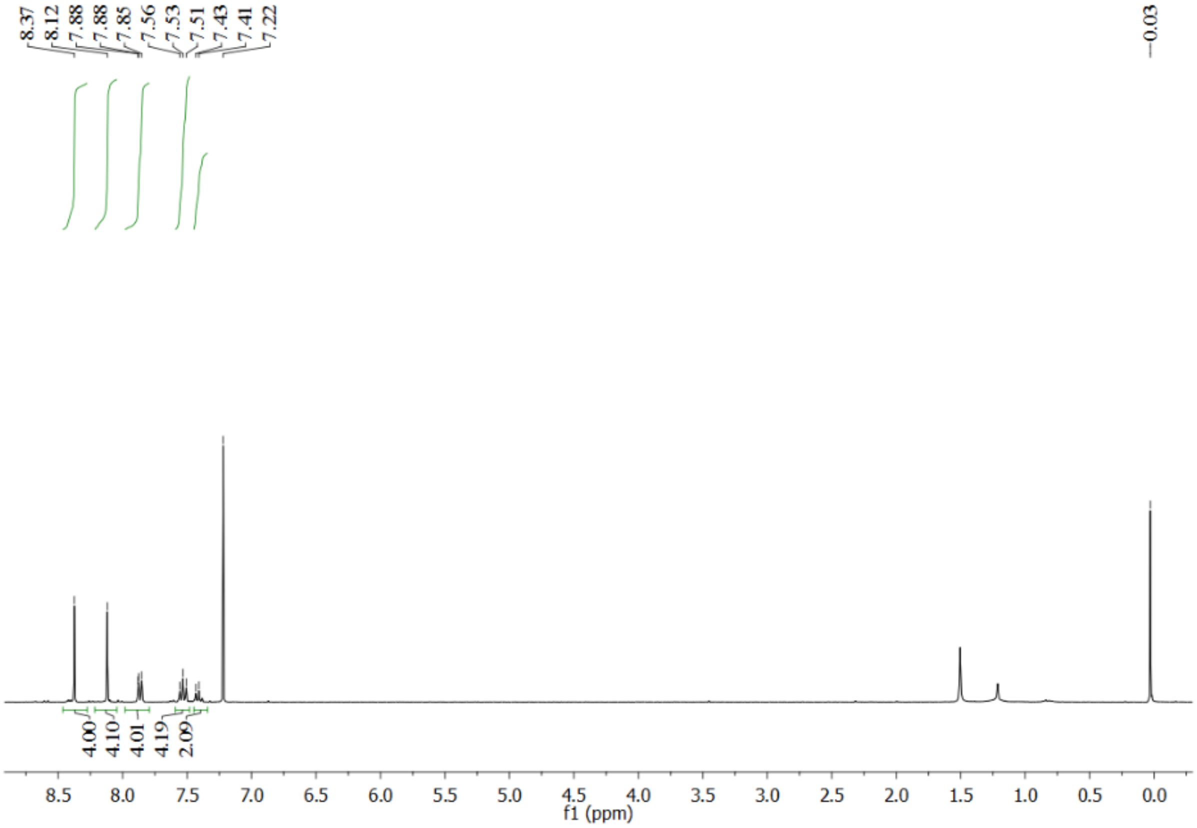

Figure S4. ^{1}H NMR spectrum of compound **4** (300 MHz, $CDCl_3$, rt).

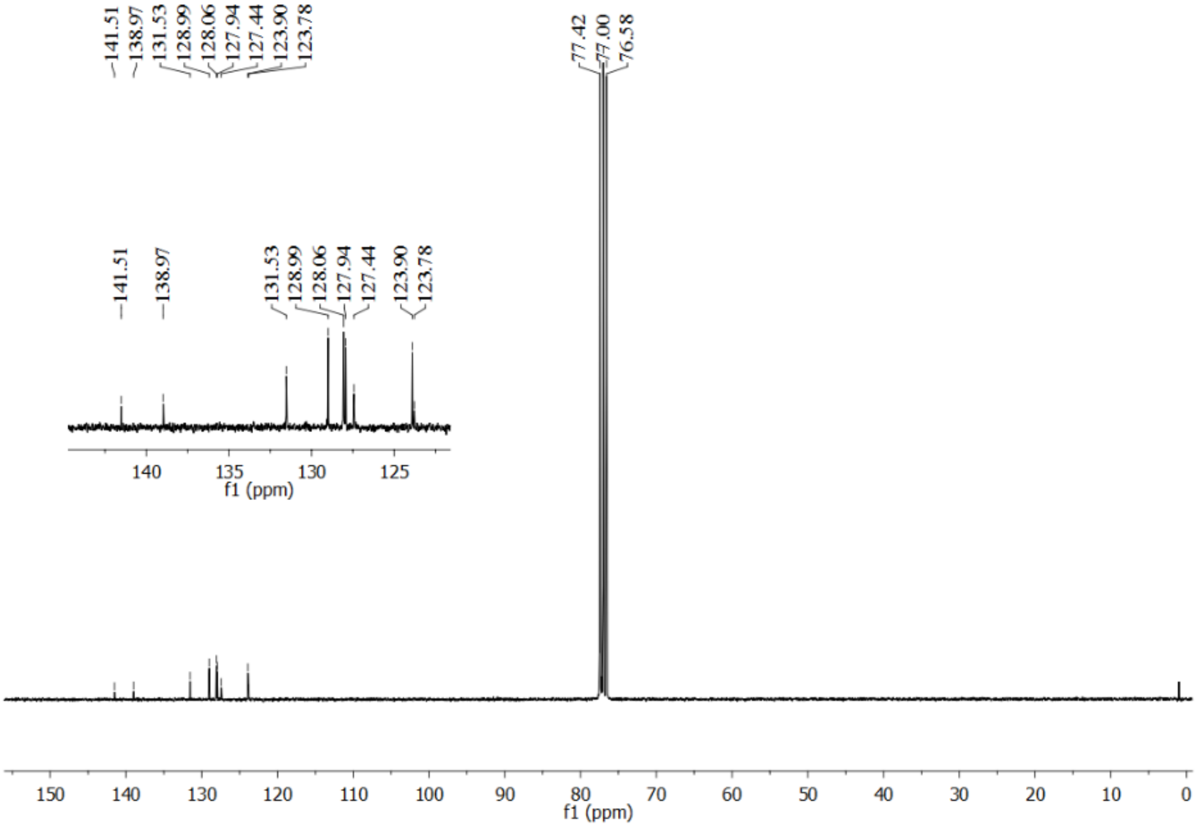

Figure S5. ^{13}C NMR spectrum of compound **4** (75 MHz, $CDCl_3$, rt).

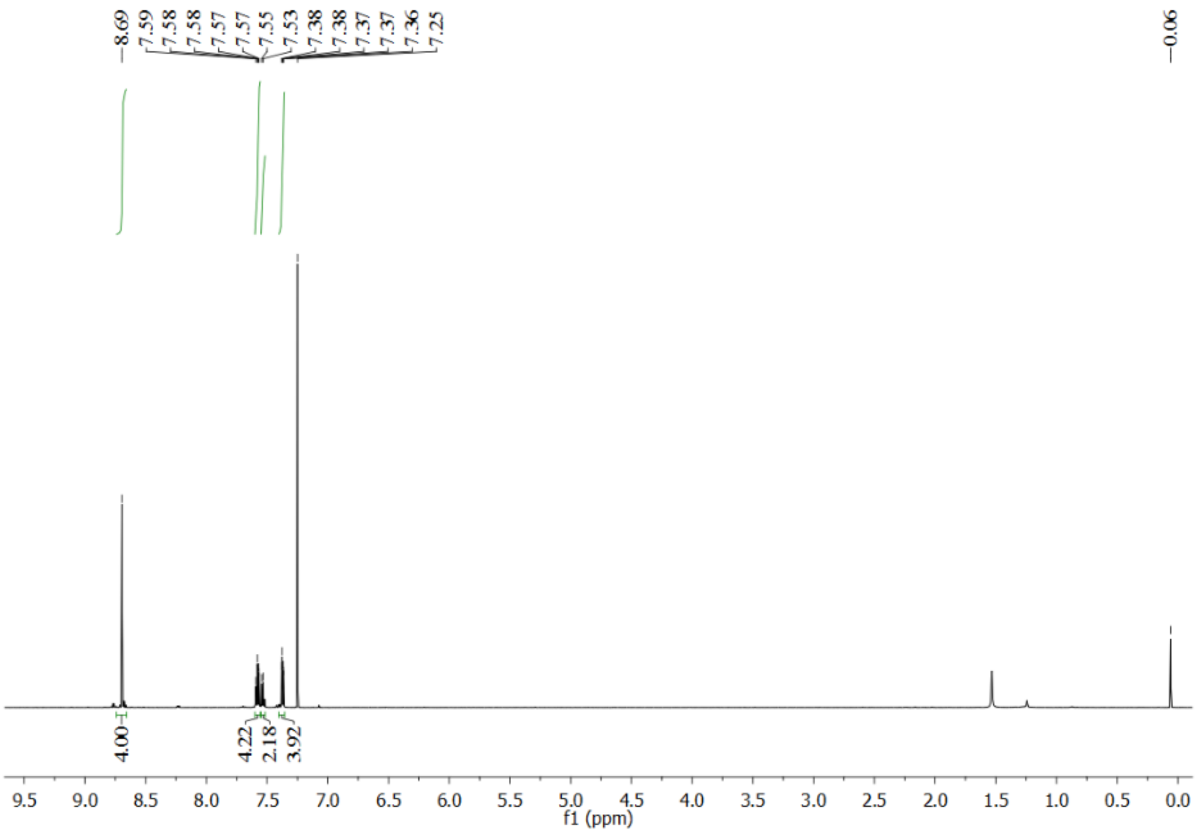

Figure S6. ^{1}H NMR spectrum of compound **5** (600 MHz, $CDCl_3$, rt).

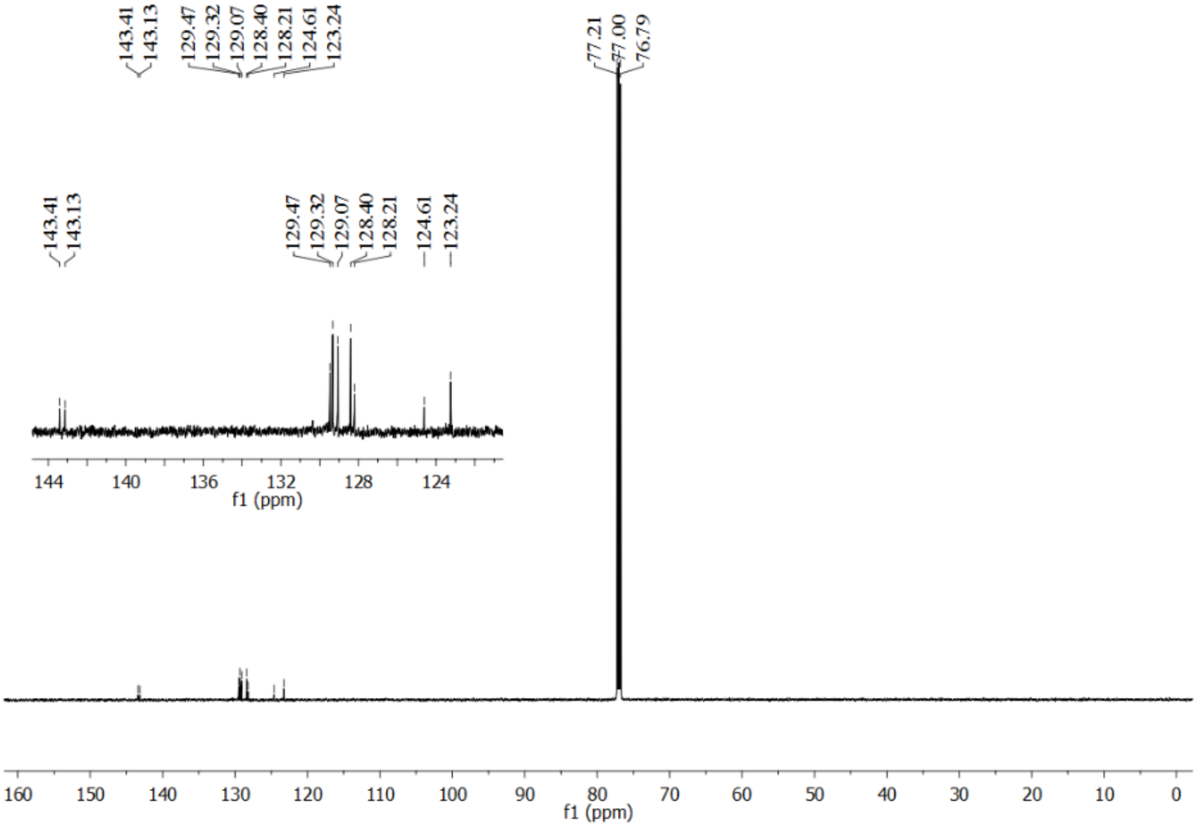

Figure S7. ^{13}C NMR spectrum of compound **5** (125 MHz, $CDCl_3$, rt).

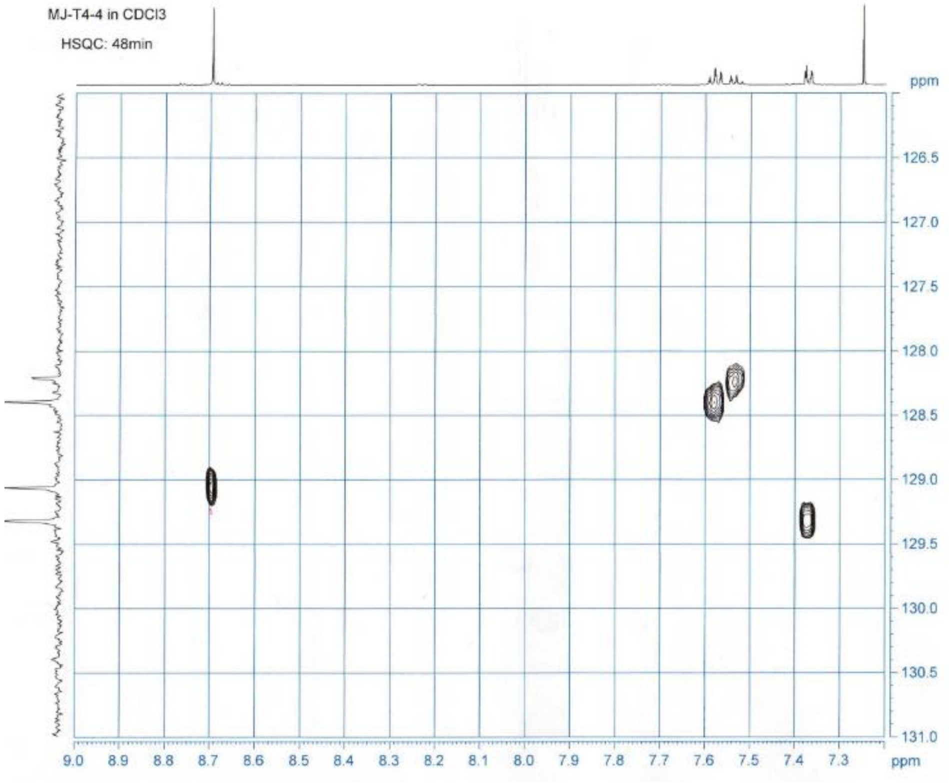

Figure S8. HSQC spectrum (region) of compound **5** ($CDCl_3$, rt).

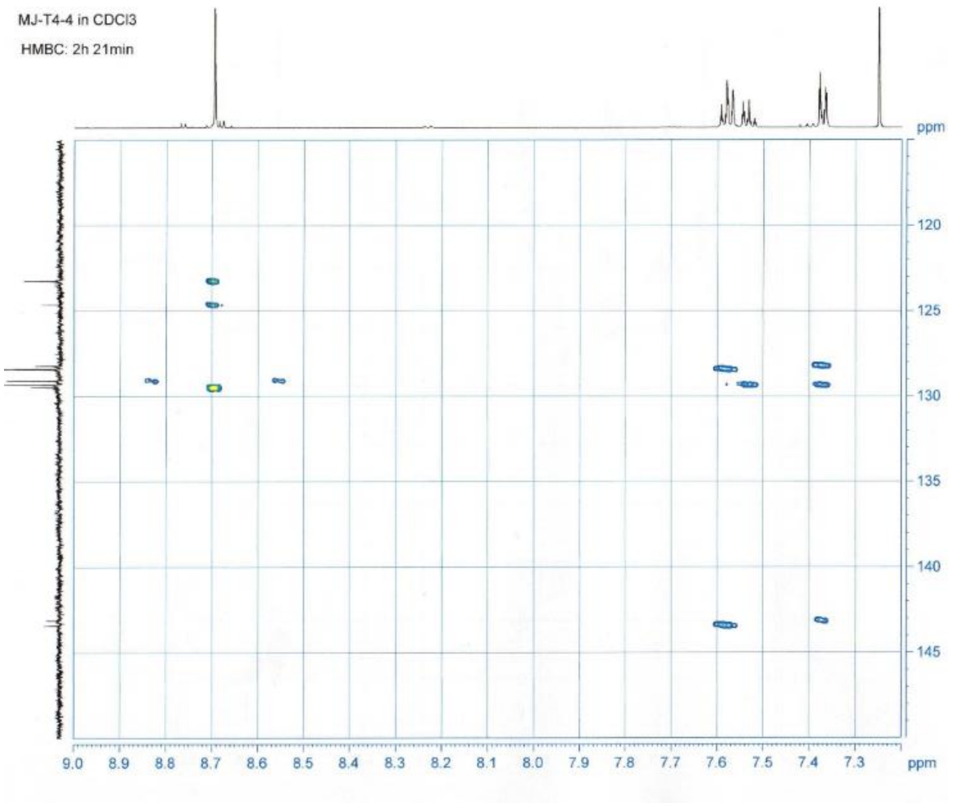

Figure S9. HMBC spectrum of compound **5** ($CDCl_3$, rt).

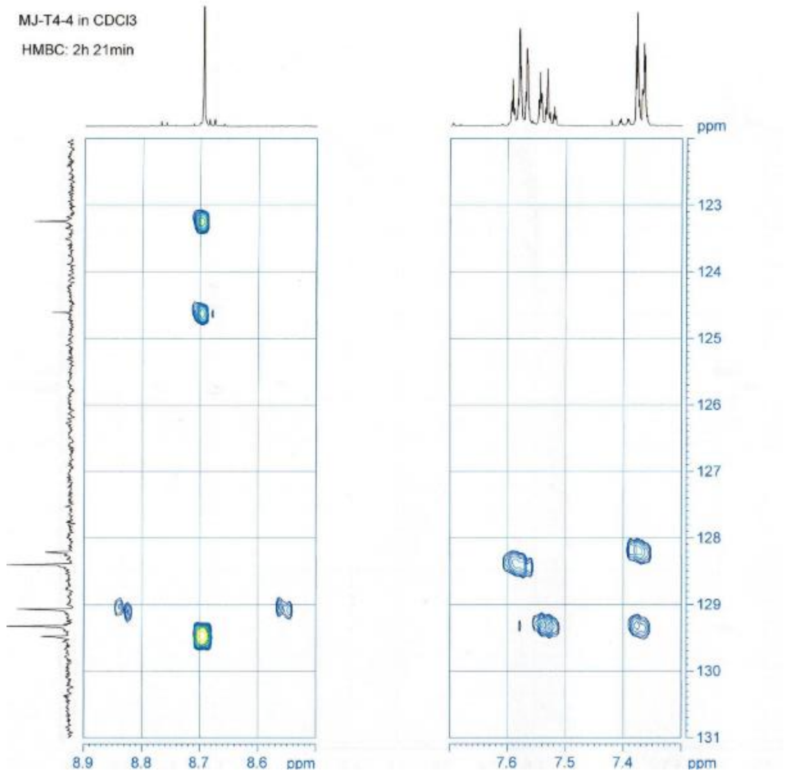

Figure S10. HMBC spectrum (region) of compound **5** ($CDCl_3$, rt).

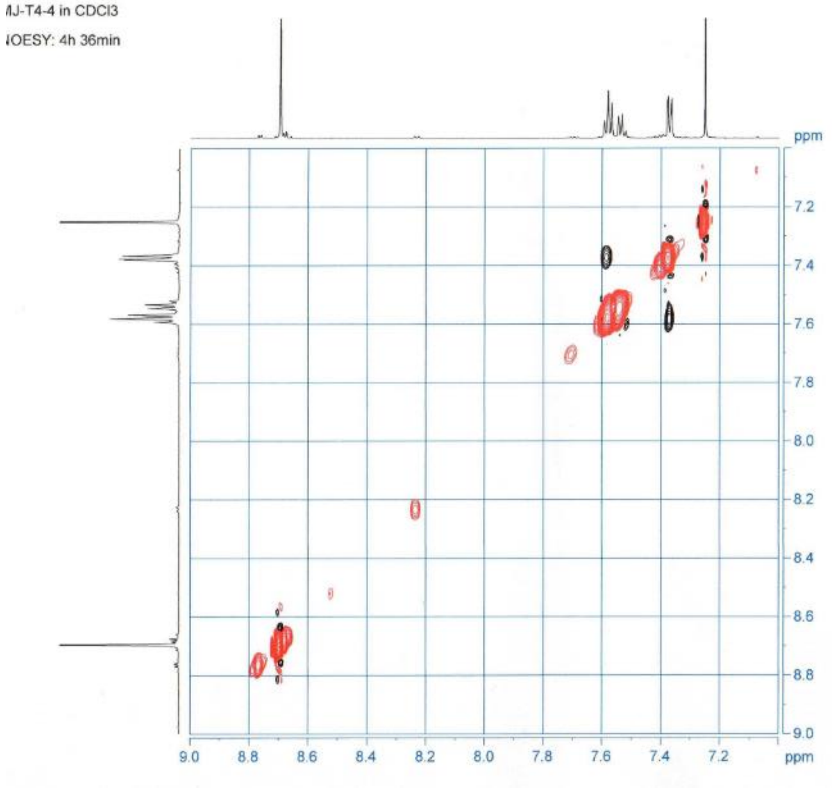

Figure S11. NOESY spectrum (region) of compound **5** ($CDCl_3$, rt).

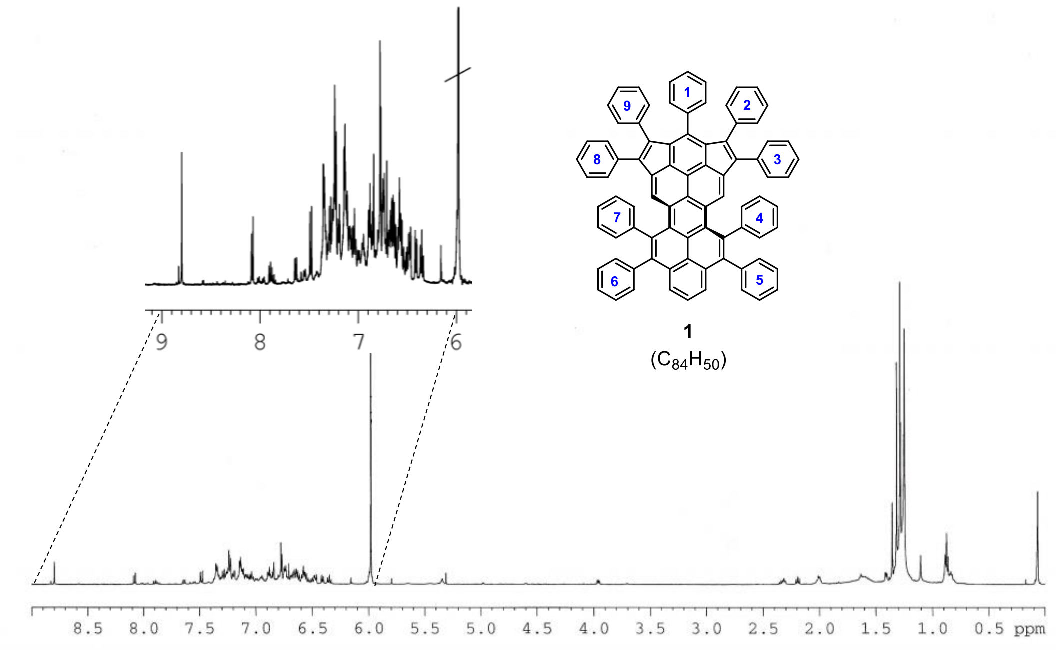

Figure S12. ^{1}H NMR spectrum of **1** ($C_2D_2Cl_4$, 30°C).

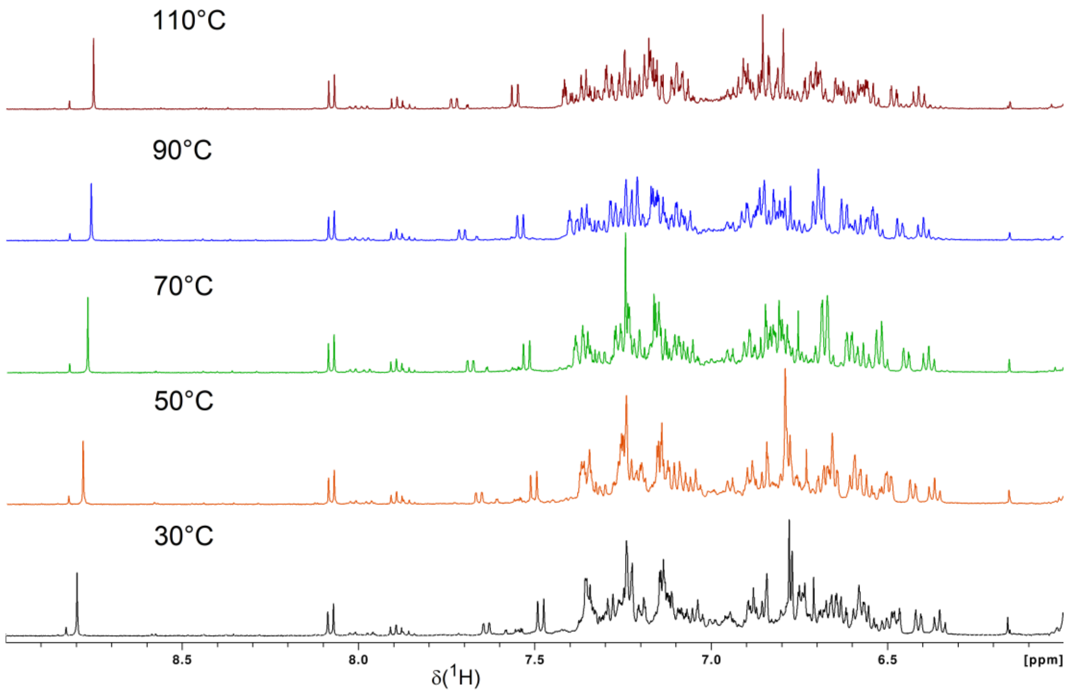

Figure S13. ^{1}H NMR spectrum (region) of **1** at different temperatures ($C_2D_2Cl_4$).

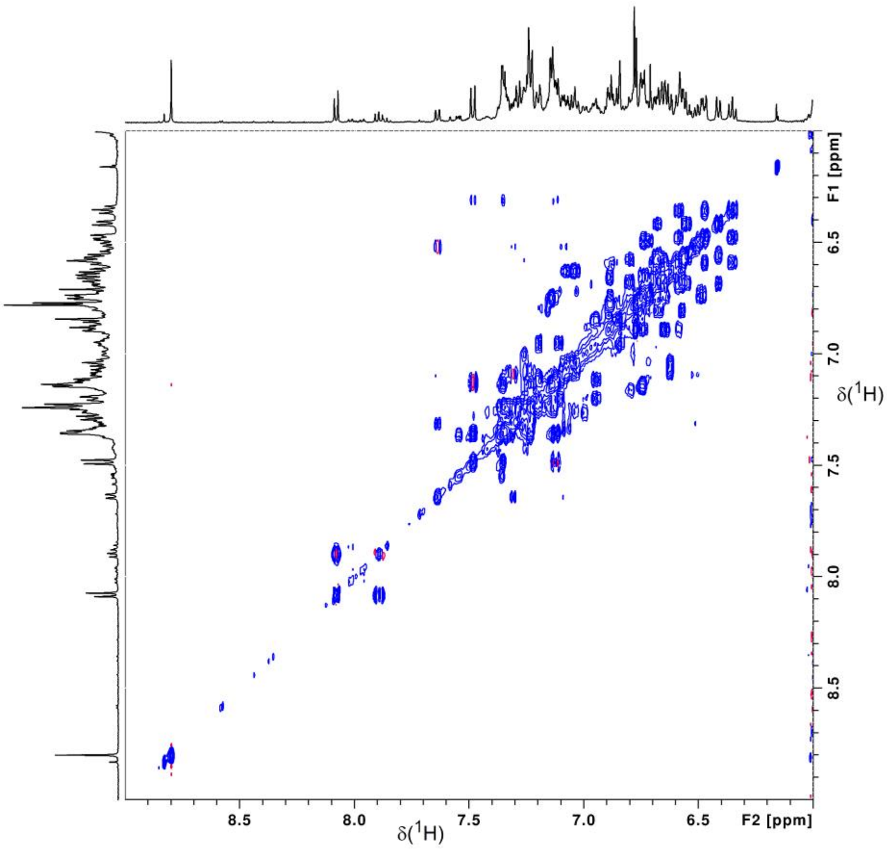

Figure S14. TOCSY spectrum (region) of **1** ($C_2D_2Cl_4$, 30°C).

5. MALDI-TOF mass spectra

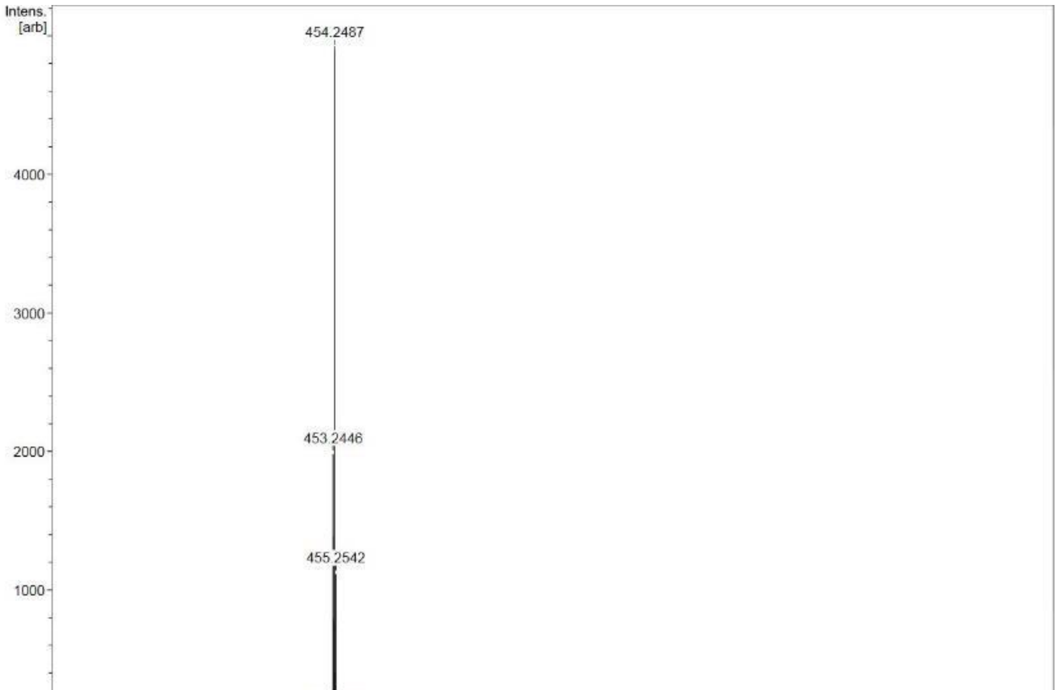

Figure S15. MALDI-TOF mass spectrum of compound **3**.

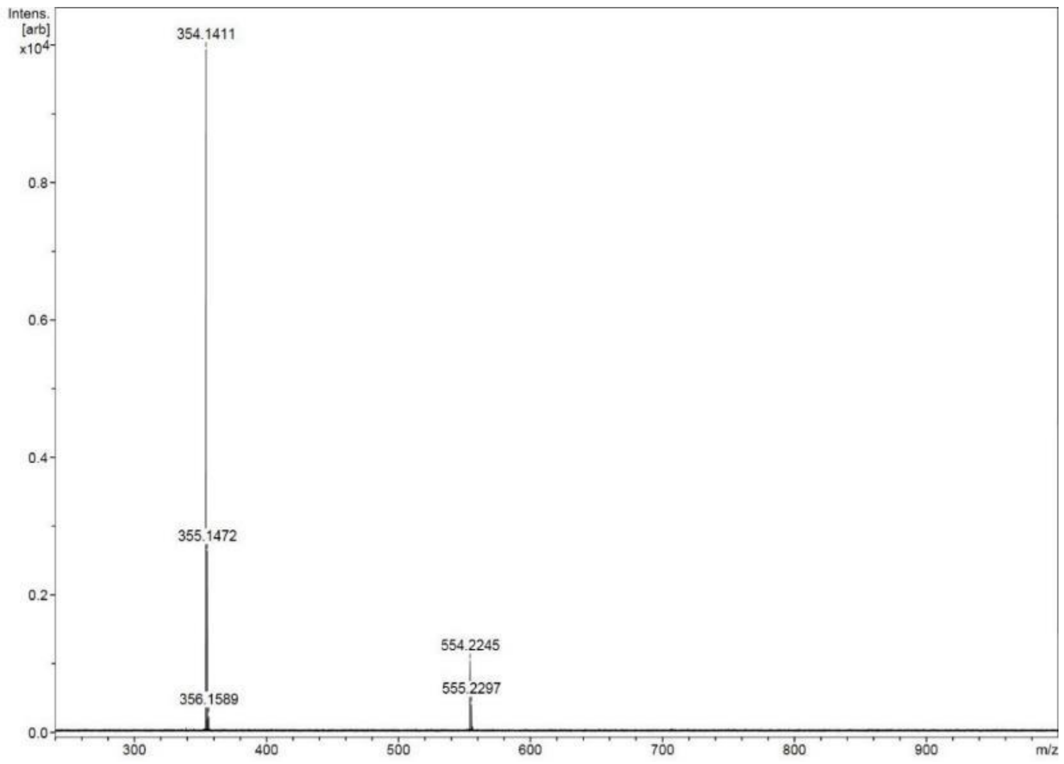

Figure S16. MALDI-TOF mass spectrum of compound **4**.

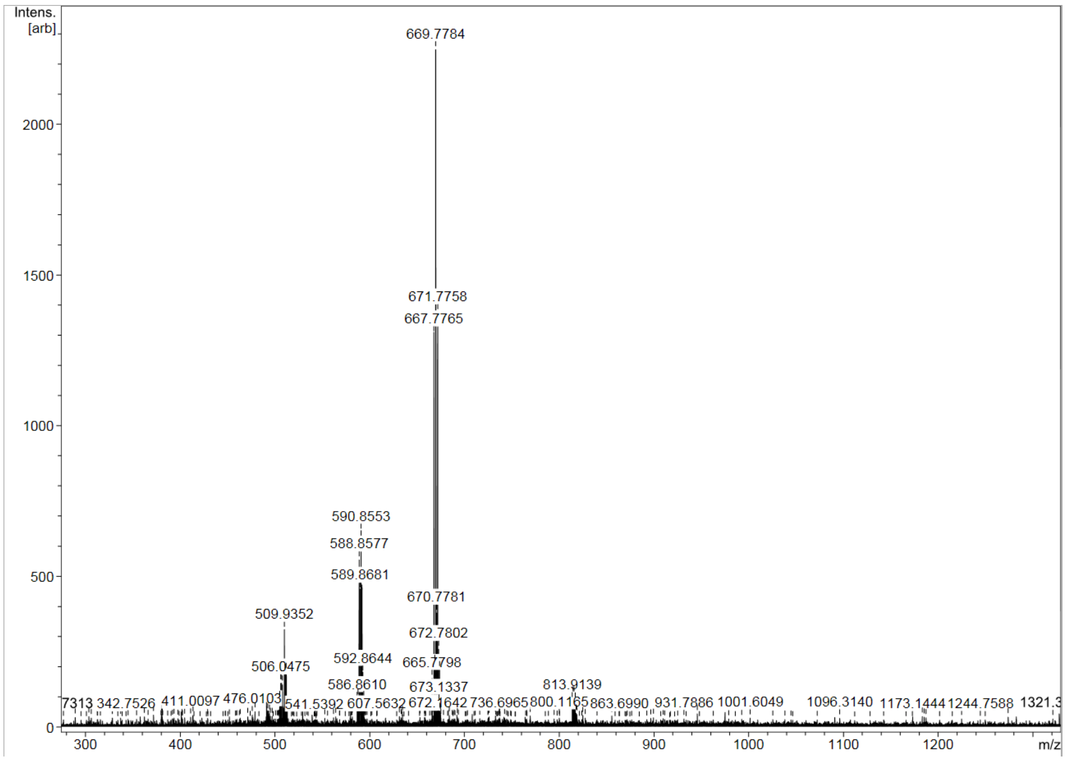

Figure S17. MALDI-TOF mass spectrum of compound **5**.

6. References

[1] D. Chercka, S.-J. Yoo, M. Baumgarten, J.-J. Kim, K. Müllen, *J. Mater. Chem. C* **2014**, *2*, 9083-9086.

[2] Y. Qiao, J. Zhang, W. Xu, D. J. T. Zhu, *Tetrahedron* **2011**, *67*, 3395-3405.

[3] X. Feng, J.-Y. Hu, F. Iwanaga, N. Seto, C. Redshaw, M. R. J. Elsegood, T. Yamato, *Org. Lett.* **2013**, *15*, 1318-1321.

[4] Gaussian 09, Revision D.01, M. J. Frisch, G. W. Trucks, H. B. Schlegel, G. E. Scuseria, M. A. Robb, J. R. Cheeseman, G. Scalmani, V. Barone, B. Mennucci, G. A. Petersson, H. Nakatsuji, M. Caricato, X. Li, H. P. Hratchian, A. F. Izmaylov, J. Bloino, G. Zheng, J. L. Sonnenberg, M. Hada, M. Ehara, K. Toyota, R. Fukuda, J. Hasegawa, M. Ishida, T. Nakajima, Y. Honda, O. Kitao, H. Nakai, T. Vreven, J. A. Montgomery, Jr., J. E. Peralta, F. Ogliaro, M. Bearpark, J. J. Heyd, E. Brothers, K. N. Kudin, V. N. Staroverov, R. Kobayashi, J. Normand, K. Raghavachari, A. Rendell, J. C. Burant, S. S. Iyengar, J. Tomasi, M. Cossi, N. Rega, J. M. Millam, M. Klene, J. E. Knox, J. B. Cross, V. Bakken, C. Adamo, J. Jaramillo, R. Gomperts, R. E. Stratmann, O. Yazyev, A. J. Austin, R. Cammi, C. Pomelli, J. W. Ochterski, R. L. Martin, K. Morokuma, V. G. Zakrzewski, G. A. Voth, P. Salvador, J. J. Dannenberg, S. Dapprich, A. D. Daniels, Ö. Farkas, J. B. Foresman, J. V. Ortiz, J. Cioslowski, and D. J. Fox, Gaussian, Inc., Wallingford CT, **2009**.

[5] (a) Z. Chen, C. S. Wannere, C. Corminboeuf, R. Puchta, P. v. R.Schleyer, *Chem. Rev.* **2015**, *105*, 3842-3888. (b) H. Fallah-Bagher-Shaidaei, S. S. Wannere, C. Corminboeuf, R., Puchta, P. v. R. Schleyer, *Org. Lett.* **2006**, *8*, 863-866.
